# From emergence to recoverability: a sociotechnical control trajectory theory of HIT-related risk

**DOI:** 10.3389/fdgth.2026.1811981

**Published:** 2026-07-16

**Authors:** Md Shafiqur Rahman Jabin

**Affiliations:** eHealth Institute, Department of Medicine and Optometry, Linnaeus University, Kalmar, Sweden

**Keywords:** digital health, HIT safety, resilience concepts, risk evolution, sociotechnical systems

## Abstract

Health information technology (HIT) is deeply embedded in contemporary clinical practice, yet incident-based research continues to show that digital systems can introduce new and sometimes widespread risks to patient safety. While prior studies have been effective in classifying types of HIT-related problems, they offer limited explanation of how such risks emerge, remain unnoticed, spread across sociotechnical systems, and ultimately lead to harm—or are successfully contained. This article advances the Sociotechnical Control Trajectory Theory, a theory-forward framework that conceptualises HIT-related risk as a dynamic process of control degradation and recovery within routine clinical practice. Rather than treating HIT incidents as isolated events or static failures, the theory explains how risk evolves over time through interactions between digital systems, clinical workflows, and organisational responses. The framework specifies four recurring phases, i.e., risk emergence, risk hiddenness, risk propagation, and recoverability or loss of control, and identifies four cross-cutting control capacities that shape outcomes at every phase: observability, interpretability, coordinated response, and system margin. The contribution of the framework lies in integrating existing sociotechnical, resilience, and systems-control perspectives into a temporally structured explanatory model specifically focused on the evolution of HIT-related risk within digitally interconnected clinical environments. To support empirical application, the article derives a set of testable propositions and proposes indirect sociotechnical indicators that may facilitate earlier recognition of emerging control degradation through incident reports, system logs, ethnographic methods, and mixed-methods designs. By shifting attention from *post hoc* incident description to the dynamic mechanisms through which HIT-related risks develop and unfold, the Sociotechnical Control Trajectory Theory provides a foundation for more proactive safety monitoring, system design, and organisational governance in increasingly complex digital health environments.

## 1 Introduction

Over the past two decades, health information technology (HIT) has become deeply embedded in clinical work, reshaping how information is generated, communicated, and acted upon. Electronic patient records, e-prescribing systems, medical imaging platforms, and patient monitoring technologies are now essential to routine care delivery (1–4). At the same time, a substantial body of incident-based research has demonstrated that HIT can introduce new risks to patient safety, including workflow disruption, delays in care, incorrect clinical decisions, and harm to multiple patients simultaneously (5–8).

Existing studies, including large-scale incident analyses, thematic classifications, and in-depth case reports, have been effective in identifying *what* goes wrong with HIT systems. Recurring problem types include software functionality failures, system configuration errors, interoperability breakdowns, and unintended consequences of system upgrades or security patching (5, 9–11). These studies have also highlighted that such problems frequently affect not only individual patients but entire workflows, departments, or organisations, sometimes over extended periods (2).

However, a persistent gap remains. Incident classifications and case descriptions tend to be retrospective and static: they catalogue events after harm or disruption has occurred, but they offer limited explanation of the *dynamic processes* through which HIT-related risks arise, remain unnoticed, and spread across sociotechnical systems before becoming visible (12). Importantly, the present theory does not assume that latent risks are directly observable prior to harm. Rather, it proposes that emerging risks may be inferred indirectly from weak sociotechnical signals and patterns of system degradation observable during routine work. As noted in prior work, the manifestations of HIT problems are often obvious, while the underlying mechanisms remain obscure, a hallmark of complex sociotechnical systems (2).

This article addresses that gap by advancing a theory-forward explanation of HIT-related risk that synthesises sociotechnical, resilience, and systems-control perspectives into a temporally structured framework focused on how organisational control over HIT-related risk degrades and recovers within routine clinical practice. Rather than introducing new incident data or analytic methods, we synthesise established empirical findings into a Sociotechnical Risk Trajectory Theory that explains how HIT-related risks move through routine clinical practice. The theory complements existing classification systems and incident analyses by focusing on mechanisms and dynamics of control degradation and recovery, rather than solely on categories, frequencies, or *post hoc* event descriptions.

A specific theoretical framework for HIT-related risk is necessary because digitally interconnected clinical systems exhibit several characteristics that distinguish them from many conventional organisational safety problems. These include the rapid scalability of errors across multiple patients and services, interoperability-driven propagation across systems, temporal persistence of latent digital risk conditions, dependence on digital representations of clinical work, and the capacity for risks to remain hidden despite apparently normal operational performance (13–16). Existing organisational management and safety frameworks provide important general approaches to governance and risk management, but may not explicitly address the dynamic sociotechnical behaviour of HIT-enabled clinical environments.

## 2 Conceptual foundations

### 2.1 HIT-related risk as an emergent sociotechnical property

HIT-related risk does not reside solely in software code, hardware components, or user behaviour. Instead, it emerges from interactions between digital systems, clinical workflows, organisational arrangements, and human adaptation. This sociotechnical view is consistent with patient safety theory and with findings from incident analyses showing that similar technical problems can have widely different consequences depending on context, timing, and organisational response (2, 17).

Across multiple studies, risks have been shown to arise during routine activities such as system upgrades, configuration changes, data transfer between systems, or everyday workarounds adopted to maintain productivity (11, 12, 18). These observations suggest that HIT risk arises from normal system operation, not solely from exceptional failure.

### 2.2 What prior incident research shows—and does not explain

Existing incident-based research on HIT-related safety has consistently identified three recurring patterns. First, HIT-related problems often emerge from multiple interacting points across clinical workflows rather than from a single identifiable failure source (2). Second, the consequences of HIT-related problems are frequently separated in time from the initiating condition, such as a system upgrade, configuration change, or interoperability issue (9–11). Third, recovery is variable; some risks are successfully contained through workarounds, contingency planning, or organisational adaptation, whereas others propagate and affect multiple patients, workflows, or services (7, 8).

While incident classification systems and case analyses have been effective in documenting these patterns, they offer only limited explanations of the mechanisms by which risks remain hidden, spread across sociotechnical systems, or become recoverable. These unresolved questions motivate the development of the present theoretical framework.

Taken together, prior incident-based studies have consistently identified patterns of distributed emergence, delayed recognition, cross-workflow effects, and variable recovery in HIT-related safety events (2, 7–11). The present theory builds upon these observations by providing a coherent explanatory framework for understanding how such patterns arise and interact over time.

## 3 Conceptualising HIT-related risk (definitional foundation)

This section defines **what HIT-related risk is** and clarifies its core properties. It does **not** explain how risk evolves over time; that work is intentionally deferred to Section 4.

### 3.1 Defining HIT-related risk

In this article, HIT-related risk is defined as the potential for patient harm, clinical disruption, or loss of safe care delivery arising from misalignment between digital representations of care and clinical reality. Digital representations include patient data, system states, alerts, automated actions, and workflow logic encoded within health information technologies (19–21).

Crucially, HIT-related risk is not synonymous with technical failure, software defects, or user error. Instead, it is a sociotechnical property that emerges from interactions between digital systems, clinical work practices, organisational arrangements, and external constraints. As a result, HIT-related risk can exist even when systems appear to be functioning as designed, and care continues without immediate adverse outcomes (22–25).

This definition aligns with incident-based findings showing that many HIT-related problems arise during routine system use, configuration, or maintenance, rather than during exceptional breakdowns.

### 3.2 Core properties of HIT-related risk

HIT-related risk exhibits several defining properties that distinguish it from many traditional patient safety hazards.

First, HIT-related risk is often latent. Latency refers to the fact that risk conditions may exist within sociotechnical systems without being recognised or detected. Risks may remain hidden because system states, workflow dependencies, or emerging anomalies are not readily observable, even when they are already influencing clinical work (26–28).

Second, HIT-related risk is frequently non-local. The location at which a risk originates may differ substantially from where its effects are experienced (29–31). For example, a configuration decision, software update, or interoperability failure may originate in one organisational or technical domain while affecting clinicians, patients, or workflows elsewhere (9, 11).

Third, HIT-related risk is scalable. Because digital systems rely on shared data objects, automation, and interoperability, a single risk condition can affect multiple patients, clinicians, or services simultaneously (30, 32, 33). This property helps explain why HIT-related incidents often involve widespread disruption rather than isolated events.

Finally, HIT-related risk is often temporally displaced. The introduction of a risk condition and the manifestation of its consequences may be separated by hours, days, or even weeks (29–31). This temporal separation can complicate attribution, investigation, and organisational learning because the initiating conditions may no longer be visible when consequences emerge (13, 14, 34).

Although related, these properties describe distinct dimensions of HIT-related risk, namely detectability, spatial distribution, scale of impact, and temporal separation between risk introduction and consequence (2, 15).

### 3.3 Why incident reports reveal risk late

Incident reporting systems play a critical role in learning from HIT-related safety problems, but they predominantly capture visible outcomes rather than the full sociotechnical context in which risk develops (22, 35). However, the theory does not reject retrospective indicators. Instead, it proposes that such indicators may also function as observable traces of broader sociotechnical trajectories that begin before major disruption or harm becomes evident.

Classification systems and thematic analyses are therefore well-suited to identifying *types* of HIT-related problems, but they are less effective at explaining how risk accumulates, persists, and escalates over time. This limitation is not a shortcoming of incident reporting itself, but a reflection of the complex and distributed nature of HIT-related risk (36–38).

To address this gap, a complementary theoretical lens is needed—one that explains what happens to risk after it is introduced into routine clinical work. That task is taken up in Section 4.

*Having defined HIT-related risk as a latent, non-local, and scalable sociotechnical property, the next section examines how organisations lose and regain control over that risk as it unfolds in routine clinical practice.*

## 4 Sociotechnical control trajectory theory

### 4.1 Overview of the sociotechnical control trajectory

The Sociotechnical Control Trajectory Theory (CTT) conceptualises health information technology (HIT)–related risk as a dynamic process of control degradation and recovery within routine clinical practice (27, 39). Rather than treating HIT incidents as isolated events or static system failures, the theory explains how risks evolve over time through interactions between digital systems, clinical workflows, and organisational responses (26, 39). The proposed trajectory was informed by recurring patterns reported in HIT-related incident investigations, including delayed risk detection, propagation across interconnected workflows, and variability in organisational recovery capacity (2, 7–11).

At the core of the theory is the idea that safe HIT-enabled care depends on an organisation's ability to maintain control over digital representations of clinical work, including patient data, system states, alerts, and automated actions (28, 40, 41). Control, in this sense, refers to the organisational capacity to maintain alignment among digital systems, clinical workflows, human interpretation, and operational decision-making, ensuring that what digital systems represent, process, and communicate remains consistent with clinical reality and intent (39, 40, 42). When this alignment erodes, risk emerges even if care continues without immediate disruption (26, 29, 43).

The theory proposes that HIT-related risks typically follow a **four-phase trajectory**:

1. **Risk emergence**, in which sociotechnical misalignments are introduced into otherwise functioning systems (2, 31);
2. **Risk hiddenness**, in which those misalignments remain latent due to limited observability, adaptation, or fragmented responsibility (2, 44);
3. **Risk propagation**, in which latent risks spread across workflows, systems, patients, or time (2, 15); and
4. **Recoverability**, in which organisations actively attempt to detect, interpret, contain, and regain control over emerging or propagating risk conditions. Where recoverability fails, trajectories may culminate in loss of control, patient harm, or widespread disruption (2, 17).

**Figure 1:**
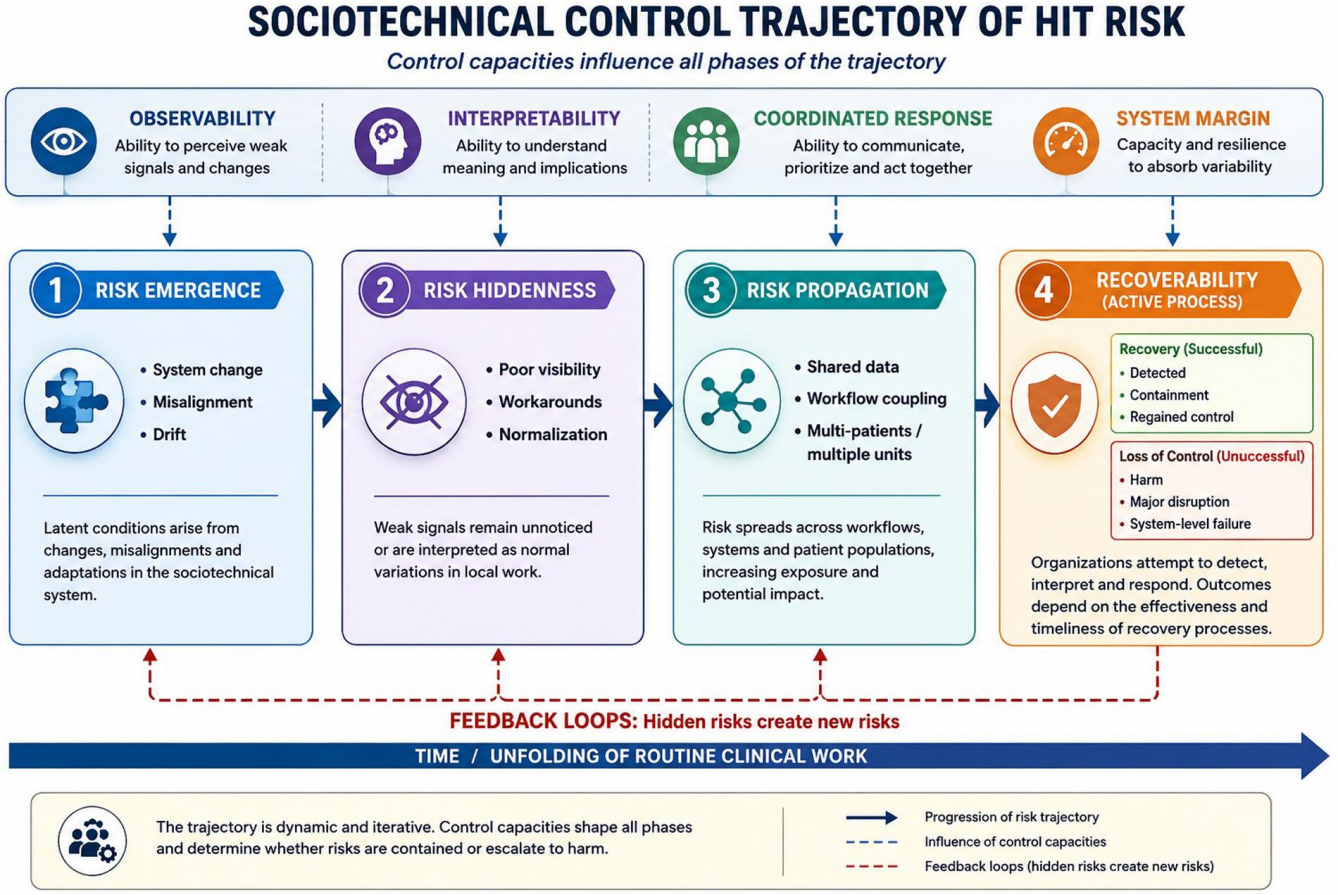
Sociotechnical control trajectory theory of HIT-related risk. The figure illustrates how health information technology (HIT)-related risks evolve through four interrelated phases: risk emergence, risk hiddenness, risk propagation, and recoverability. Across all phases, organisational control is shaped by four cross-cutting sociotechnical control capacities: observability, interpretability, coordinated response, and system margin. The trajectory is dynamic and iterative, with feedback loops allowing unresolved or hidden risks to generate new risk conditions over time. Recoverability represents an active organisational process through which risks may be contained or control regained, whereas unsuccessful recoverability may culminate in loss of organisational control, operational disruption, or patient harm.

These phases are illustrated in Figure 1, which depicts the control trajectory as an unfolding process rather than a linear cause–and–effect chain. The phases may overlap, recur, or accelerate depending on local conditions such as system coupling, workload, and governance arrangements.

**Figure 2:**
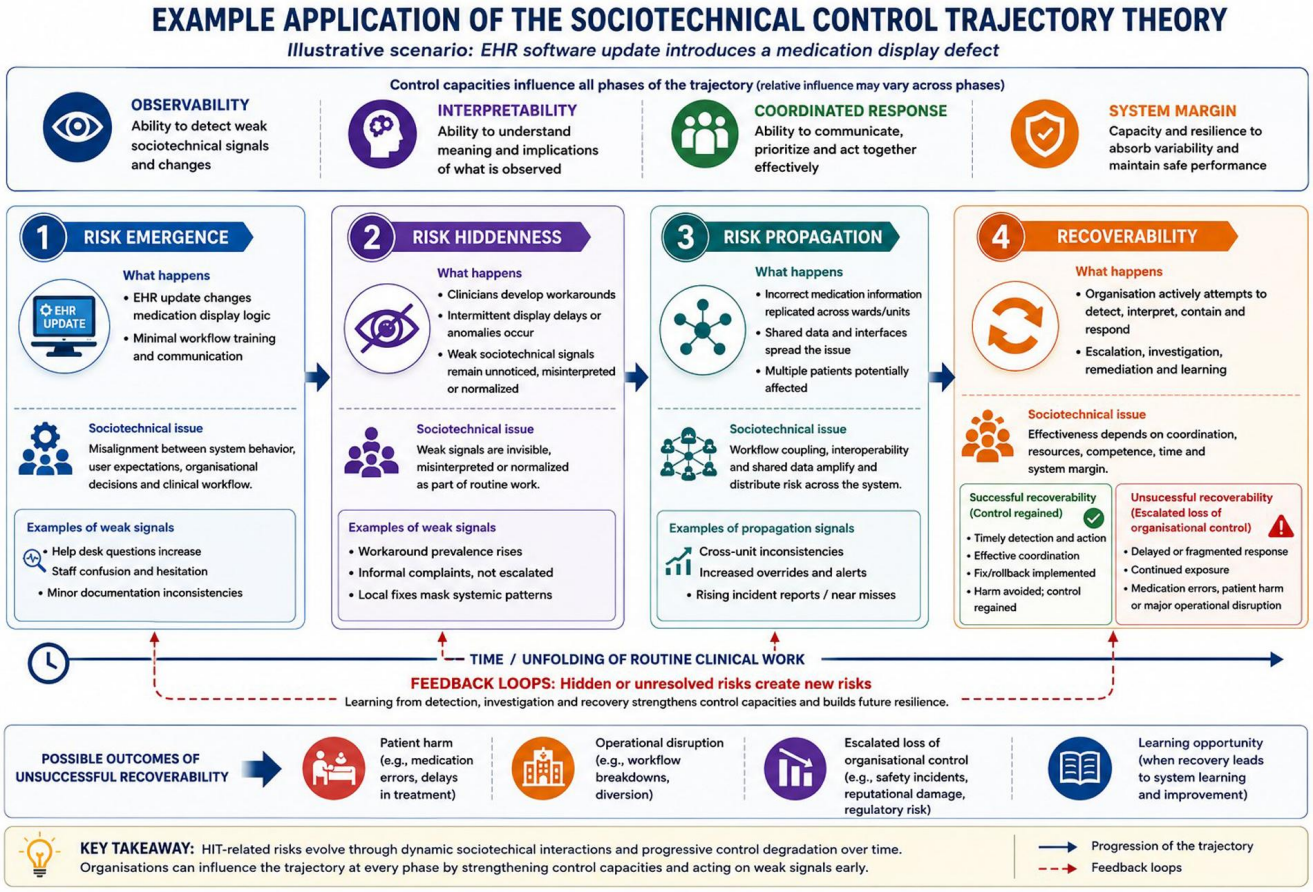
Example application of the sociotechnical control trajectory theory using an illustrative EHR software update scenario. The figure demonstrates how an HIT-related risk can evolve through emergence, hiddenness, propagation, and recoverability within routine clinical practice, while illustrating the sociotechnical interactions and control capacities that shape outcomes across the trajectory.

Importantly, the trajectory is shaped by four cross-cutting control capacities that influence outcomes at every phase: observability, interpretability, coordinated response, and system margin (39). Variation in these capacities helps explain why similar technical issues can lead to minor inconvenience in one setting and major patient safety events in another (15, 39). However, control degradation within the trajectory is not treated as a purely technical phenomenon. Across all phases, control is shaped by interactions between technologies, frontline adaptations, organisational structures, communication pathways, and governance practices within routine clinical work (15, 45).

Sections 4.2–4.5 elaborate each phase of the control trajectory in turn, specifying the sociotechnical mechanisms through which HIT-related risks are generated, concealed, amplified, and either recovered from or allowed to escalate.

### 4.2 Risk emergence: how HIT-related risks are generated

In the Sociotechnical Control Trajectory Theory, risk emergence refers to the initial loss of alignment between digital systems and the realities of clinical work. Importantly, emergence does not imply immediate failure or harm. Instead, risk arises when digital representations of care no longer reliably align with clinical intentions, workflows, or system states, while work continues under the assumption of normal operation (36, 46–48).

Across incident-based studies, risk emergence is consistently associated with routine and often well-intentioned activities, including system upgrades, software security patching, configuration changes, and the introduction of new integrations. These activities alter system behaviour, performance, or dependencies, frequently without proportional changes in training, workflow redesign, or contingency planning. The resulting misalignment is sociotechnical in nature: it arises not from technology alone, but from interactions among technology, clinical interpretation, communication practices, organisational decision-making, workflow adaptation, and operational constraints (2, 9, 10, 45).

A key mechanism at this stage is configuration drift, i.e., the gradual divergence between how systems are configured or updated and how clinical work is actually performed (18, 49). Because many HIT systems are highly parameterised and interconnected, small configuration changes can introduce latent risk conditions that remain invisible during testing or commissioning but become consequential under real-world workload and time pressure. This helps explain why incidents related to system upgrades or patching often occur in otherwise stable environments and why similar technical changes can produce markedly different outcomes across organisations (2, 5, 50).

Crucially, risk emergence is rarely experienced by frontline staff as an “error” or “failure”. Instead, it is embedded in normal work, masked by temporary adaptations, and tolerated as part of everyday operational complexity (2, 17, 51).

### 4.3 Risk hiddenness: why risks remain latent and unrecognised

Following emergence, HIT-related risks frequently enter a phase of hiddenness, during which the organisation progressively loses visibility of the risk condition. Hiddenness is not simply a lack of reporting; it is an active sociotechnical process through which signals of risk are attenuated, delayed, fragmented, or normalised (28, 40, 52).

One central mechanism is attenuated observability. Many HIT systems provide limited transparency into their internal state, dependencies, or downstream effects. For example, staff may be unable to see whether data are incomplete, delayed, or mis-synchronised across systems, or whether a software patch has fully propagated. As a result, early warning signs—such as subtle performance degradation, intermittent errors, or increased manual work—are interpreted as local nuisances rather than indicators of systemic risk (29, 53, 54). These interpretations are socially mediated and shaped by local workflow culture, communication norms, competing operational priorities, and prior experiences with system instability (40, 55).

A second mechanism is normalisation through adaptation. Frontline clinicians and technical staff routinely develop workarounds to maintain care delivery despite minor disruptions. While these adaptations demonstrate resilience, they also reduce the likelihood that emerging risks are escalated or formally recognised (21, 22). Over time, degraded system performance can become “the new normal,” further obscuring the underlying loss of control (15, 30, 40).

Hiddenness is reinforced by organisational fragmentation. Responsibility for HIT systems is often distributed across clinical, technical, and managerial domains, each with partial and non-overlapping perspectives. This fragmentation means that no single actor has a complete view of how local issues might combine or escalate. Consequently, risks may persist for extended periods before being formally identified—often only after significant disruption or harm occurs (15, 38, 55).

### 4.4 Risk propagation: how risks spread across workflows and systems

When hidden risks interact with tightly coupled sociotechnical structures, they may begin to propagate. Risk propagation refers to the process by which a locally originating HIT-related risk spreads across patients, workflows, departments, organisations, or time (39, 56).

Digital systems are particularly prone to propagation because they rely on shared data objects, automated processes, and interoperability links. Once incorrect, delayed, or degraded information enters a system, it can be rapidly reused, copied forward, or acted upon by downstream processes with minimal human verification. This creates conditions in which a single initiating issue can affect multiple patients or clinical activities, sometimes simultaneously (16, 27, 30).

Propagation is also shaped by sociotechnical workflow coupling, in which technical interdependencies interact with communication structures, coordination practices, and distributed clinical responsibilities (56, 57). Incident analyses consistently show that problems originating in one part of the system (e.g., imaging or monitoring) can have downstream effects on diagnosis, treatment decisions, or patient flow, even when the original issue is no longer visible (29, 31, 58).

A defining feature of propagation in HIT-related risk is temporal extension. Unlike many traditional safety events, digital risks can persist and re-emerge over days or weeks, especially when underlying causes are not fully understood or addressed. This explains why some HIT incidents result in prolonged operational disruption or affect large patient populations before being resolved (39, 40).

### 4.5 Recoverability and loss of control: why some risks are contained, and others cause harm

The final phase of the control trajectory concerns recoverability, an active sociotechnical process through which organisations attempt to detect, interpret, contain, and respond effectively to emerging or propagating risk. Recoverability determines whether control is regained or whether the trajectory culminates in patient harm, widespread disruption, or both (26, 29, 39).

Recoverability depends less on the intrinsic quality of software and more on sociotechnical control capacities, including:

- the ability to detect anomalies in system behaviour,
- the interpretability of signals indicating that something is wrong,
- the availability of coordinated response pathways, and
- the presence of system margin, such as contingency plans, backup systems, and staffing flexibility (39, 40).

Incident-based evidence shows that organisations with robust contingency planning and clear escalation pathways are more likely to contain HIT-related risks before harm occurs, even when technical problems are significant. Conversely, where recovery mechanisms are weak or absent, relatively modest technical issues can escalate into major safety events (30, 32).

Importantly, recoverability does not guarantee successful recovery once harm begins to manifest. Where recoverability processes are delayed, fragmented, or ineffective, trajectories may culminate in irreversible loss of organisational control. In some cases, delayed recognition or fragmented responsibility can lead to an irreversible loss of control, particularly when risks have already propagated across multiple systems or patients (29, 39). This highlights why recoverability must be understood as an active, time-sensitive process rather than a passive system property.

Together, these four phases, i.e., emergence, hiddenness, propagation, and recoverability, form a sociotechnical control trajectory through which organisations progressively lose, attempt to maintain, and potentially regain control over HIT-related risk. In the next section, we translate this theory into testable propositions that can guide future empirical research and proactive safety monitoring. Figure 2 presents the proposed Sociotechnical Theory of Health Information Technology–Related Risk, illustrating how interactions among sociotechnical dimensions contribute to health information technology–related risks and influence healthcare quality outcomes.

## 5 Testable propositions derived from the sociotechnical control trajectory theory

A central aim of the Sociotechnical Control Trajectory Theory is to move beyond *post hoc* explanations and to support earlier, more prospective recognition of emerging HIT-related risk conditions through indirect sociotechnical indicators that may become observable before large-scale harm occurs. The theory, therefore, yields a set of testable propositions that can be examined using incident reports, ethnographic observation, system logs, simulation studies, or mixed-method designs. These propositions are organised according to the four phases of the control trajectory.

Many of the propositions reflect patterns previously reported in HIT safety research. Their contribution lies in bringing these observations together within a unified explanatory framework that specifies how risks evolve through interconnected sociotechnical processes (2, 7–11).

Importantly, several of these propositions are consistent with observations reported in the existing HIT safety literature. The purpose of the propositions is not to imply an absence of evidence, but to organise existing and future findings within a coherent explanatory framework focused on sociotechnical control trajectories (2, 7–11).

### 5.1 Propositions related to risk emergence

**Proposition 1:** HIT-related risks are more likely to emerge following system changes (e.g., upgrades, configuration changes, or security patching) that alter system behaviour without corresponding adaptation of clinical workflows or training.

**Proposition 2:** Highly configurable HIT systems are more prone to emergent behavior when local configuration decisions are made without visibility into downstream workflow dependencies.

These propositions can be examined by comparing incident frequency and characteristics before and after system changes, and by analysing how configuration decisions are documented, communicated, and operationalised.

### 5.2 Propositions related to risk hiddenness

**Proposition 3:** HIT-related risks are more likely to remain latent in systems with low observability of internal states, data flows, or integration status.

**Proposition 4:** The likelihood that emerging HIT-related risks are formally recognised decreases as frontline staff rely more on informal workarounds.

These propositions can be tested by examining the relationship between system transparency (e.g., availability of dashboards, audit trails, system-state indicators) and time-to-detection of incidents, as well as by analysing narrative incident reports for evidence of normalisation and adaptation.

### 5.3 Propositions related to risk propagation

**Proposition 5:** HIT-related risks propagate more rapidly and broadly in systems with high workflow coupling and extensive reuse of shared digital data objects.

**Proposition 6:** Interoperability between HIT systems increases the scale of impact of latent risks when data validation and verification mechanisms are weak.

These propositions can be investigated through network-based analyses of workflows and systems, tracing how errors or disruptions spread across patients, departments, or organisations.

### 5.4 Propositions related to recoverability and loss of control

**Proposition 7:** The likelihood of successful recovery from HIT-related risk is more strongly associated with organisational control capacities (e.g., contingency planning, escalation pathways, staffing flexibility) than with the technical severity of the initiating issue.

**Proposition 8:** Delayed recognition of HIT-related risk reduces recoverability disproportionately once risk has propagated across multiple workflows or patient groups.

These propositions can be explored using multiple sources of evidence, including incident reports, system logs, ethnographic observations, simulation studies, Healthcare Failure Mode and Effects Analysis (HFMEA), and other prospective risk assessment approaches, with a focus on detection timing, response coordination, workflow adaptation, and organisational control capacities.

### 5.5 Implications for empirical research and monitoring

Together, these propositions provide a structured research agenda for examining HIT-related safety beyond incident counting or classification. The framework is therefore intended to complement existing retrospective safety methods by encouraging interpretation of incident signals as indicators of ongoing sociotechnical control trajectories rather than isolated failures. They also suggest opportunities for proactive safety monitoring, such as tracking indicators of hiddenness (e.g., workaround prevalence), propagation (e.g., cross-workflow dependencies), and recoverability (e.g., response latency).

In the following section, we discuss how the Sociotechnical Control Trajectory Theory extends existing frameworks, its implications for governance and design of digital health systems, and its limitations.

## 6 Discussion

The contribution of the Sociotechnical Control Trajectory Theory is not the identification of entirely new risk phenomena, but the integration of previously observed patterns into a temporally structured sociotechnical explanation of how HIT-related risks emerge, remain hidden, propagate, and become recoverable within routine clinical practice (2, 7–11, 15).

The Sociotechnical Control Trajectory Theory should be understood as an integrative HIT-specific explanatory framework rather than a wholly new safety paradigm. The framework synthesises concepts from sociotechnical theory, resilience engineering, systems control thinking, and patient safety research to explain how HIT-related risks evolve over time within digitally interconnected clinical environments. Its contribution lies not in introducing entirely new safety constructs, but in organising existing concepts into a coherent temporal model that explains processes of emergence, hiddenness, propagation, and recoverability.

### 6.1 Theoretical contributions beyond existing frameworks

The Sociotechnical Control Trajectory Theory advances the field of digital health safety in three important ways.

First, it shifts the analytical focus from static categories of HIT problems to dynamic processes of risk evolution. Existing frameworks, such as sociotechnical work system models and HIT incident classification systems, i.e., have been highly effective in identifying *where* and *what* types of problems occur (2). However, they offer a limited explanation of *how* risks unfold over time, particularly why similar technical issues can lead to minor inconvenience in some settings and widespread harm in others (2, 3). By conceptualising HIT-related risk as a trajectory of control degradation and recovery, the present theory explicitly accounts for time, interaction, and escalation. More specifically, the theory conceptualises safety as the maintenance, degradation, and recovery of sociotechnical control over digital clinical work, rather than as the simple presence or absence of technical failure.

Second, the theory foregrounds hiddenness and propagation as central mechanisms rather than incidental features of HIT-related incidents. Incident-based research has repeatedly shown delayed recognition, multi-patient impact, and prolonged disruption, yet these patterns are often treated as outcomes rather than processes (6–9). The control trajectory framework explains why digital risks are especially prone to remaining latent and to scaling across workflows: shared data objects, automation, and interoperability create conditions in which local problems can become systemic before they are detected.

Third, the theory elevates recoverability to a core theoretical construct. Much of the HIT safety literature implicitly assumes that detection leads to resolution (5, 6). In contrast, the control trajectory theory shows that recovery is contingent, time-sensitive, and socially organised. This aligns with resilience-oriented perspectives in patient safety but extends them by specifying how recoverability interacts with digital system characteristics and organisational control capacities. Within the theory, recoverability is conceptualised as a dynamic organisational process rather than a terminal outcome. Harm or loss of control represents a possible consequence of unsuccessful recoverability, not the phase itself.

Together, these contributions position the theory as complementary to, rather than competitive with, existing sociotechnical and classification frameworks. Classification systems remain essential for organising empirical evidence (12). Accordingly, the theory should not be interpreted as replacing retrospective safety approaches, but rather as extending them by providing a dynamic, process-oriented account of how observed signals relate to evolving sociotechnical control conditions.

### 6.2 Implications for safety monitoring, design, and governance

The Sociotechnical Control Trajectory Theory has several practical implications for improving the safety of HIT-enabled care.

For safety monitoring, the theory suggests that organisations should move beyond relying solely on harm-based indicators. Early signals of risk are more likely to appear as indicators of hiddenness and propagation, such as increasing reliance on workarounds, unexplained performance degradation, or cross-workflow inconsistencies. Monitoring these signals may allow intervention before risks escalate into reportable incidents or patient harm (2, 21, 49, 57).

For system design and implementation, the theory highlights the importance of designing for observability and interpretability. HIT systems that obscure their internal states, dependencies, or data flows undermine organisational control and increase the likelihood that emerging risks will remain hidden (16, 52). Design strategies that make system behaviour transparent, particularly during upgrades, patching, and integration, can enhance recoverability even when technical problems arise.

For governance and management, the findings underscore that control over HIT-related risk is not achieved solely through technical fixes. Organisational capacities such as contingency planning, clear escalation pathways, cross-functional coordination, and training play a decisive role in determining whether control is regained. This has implications for procurement, commissioning, and lifecycle management of HIT systems, suggesting that governance arrangements should explicitly address recovery as well as prevention (38, 59, 60).

### 6.3 Relationship to existing safety and management frameworks

The Sociotechnical Control Trajectory Theory is intended to complement, rather than replace, existing approaches to patient safety, resilience, and organisational management. Several concepts incorporated within the framework draw upon established traditions in systems thinking, resilience engineering, organisational safety, and risk management. However, the theory differs from many existing approaches in its explicit focus on the temporal evolution of sociotechnical control degradation and recovery within HIT-enabled clinical work.

The need for an HIT-specific theoretical framework arises from the distinctive properties of digitally interconnected healthcare systems. Unlike many conventional organisational risks, HIT-related risks may propagate simultaneously across patients, departments, and institutions through shared digital infrastructures, automation, and interoperability mechanisms. In addition, digital risks may persist in latent form despite apparently stable clinical operations, particularly when frontline adaptations temporarily compensate for degraded system conditions. The proposed framework was developed specifically to conceptualise these dynamic and distributed sociotechnical characteristics of HIT-related risk within routine clinical work.

For example, Failure Mode and Effects Analysis (FMEA) and Ishikawa diagrams primarily support prospective hazard identification and causal analysis, whereas the present theory focuses on how risks dynamically evolve, remain hidden, propagate across interconnected workflows, and become recoverable over time (28, 29, 39). Similarly, the Swiss Cheese Model conceptualises safety as layered organisational defences against failure, whereas the present framework emphasises the progressive degradation of sociotechnical control capacities in routine digital clinical work (28, 29).

Resilience engineering approaches strongly inform the theory, particularly regarding adaptation, monitoring, and recovery capacities (26, 40). However, the proposed framework extends these perspectives by organising HIT-related risk into a structured trajectory that explicitly links emergence, hiddenness, propagation, and recoverability within digitally interconnected healthcare environments.

The proposed theory is also closely related to frameworks that specifically address technology-induced errors and sociotechnical HIT safety. Borycki and colleagues focused on identifying where technology-induced errors originate within interactions between technologies, users, tasks, and organisational contexts (61), while Sittig and Singh emphasised the multiple sociotechnical dimensions that influence EHR-related safety (62). These perspectives help explain the origins and contributing conditions of HIT-related risk. In contrast, the Sociotechnical Control Trajectory Theory focuses on how such risks evolve over time after their introduction, including processes of hiddenness, propagation, and recoverability. Accordingly, these frameworks should be viewed as complementary: one helps explain where risks originate, whereas the present theory seeks to explain how those risks subsequently unfold within routine clinical practice.

Organisational management approaches, including Balanced Scorecard methods and change management frameworks such as Kotter's model, primarily address governance, implementation, and organisational performance improvement (63–65). In contrast, the present theory specifically addresses the sociotechnical dynamics through which HIT-related risks evolve across digital infrastructures, clinical workflows, and organisational response systems.

Accordingly, the framework is intended to complement existing HIT safety approaches by providing a temporal and process-oriented explanation of how risk conditions evolve, interact, and become observable across interconnected sociotechnical systems.

Table 1 summarises key distinctions between the proposed Sociotechnical Control Trajectory Theory and selected existing safety and organisational management frameworks

**Table 1:** Comparison of the sociotechnical control trajectory theory with selected existing safety and management frameworks.

Framework	Primary focus	Ability to address dynamic HIT-specific sociotechnical risk
Swiss Cheese Model (29)	Organisational defenses and latent failures	Informs the theory's understanding of latent conditions and layered organisational vulnerability
Failure Mode and Effects Analysis (FMEA)	Prospective hazard identification and mitigation	Complements the theory's trajectory-based interpretation of emerging risk conditions
Ishikawa (Fishbone) Diagram (29)	Root-cause categorisation	Supports causal analysis but does not explicitly model temporal risk evolution
Resilience Engineering (66)	Adaptation, monitoring, recovery, system resilience	Strongly informs the theory's concepts of recoverability, adaptation, and organisational response
Kotter Change Management Framework (64)	Organisational change implementation	Provides broader organisational perspectives relevant to HIT implementation and adaptation
Balanced Scorecard (63)	Strategic performance monitoring	Relevant to governance and organisational oversight, but not specifically focused on dynamic HIT-risk evolution
Technology-Induced Error Framework (61)	Identification of technology-induced error sources arising from interactions between technology, users, tasks, and organisational contexts	Provides insight into where HIT-related risks originate, but does not explicitly model how risks evolve, propagate, or become recoverable over time
Sociotechnical EHR Safety Framework (62)	Sociotechnical dimensions influencing EHR-related safety across technical, human, and organisational domains	Provides a comprehensive framework for understanding determinants of HIT safety, but does not explicitly describe the temporal trajectory of risk emergence, hiddenness, propagation, and recoverability
Sociotechnical Control Trajectory Theory (proposed)	Dynamic evolution of HIT-related risk through emergence, hiddenness, propagation, and recoverability	Integrates and contextualises existing sociotechnical, resilience, and systems-control perspectives within digitally interconnected clinical environments

As shown in Table 1, the proposed theory does not seek to replace existing approaches but rather to integrate sociotechnical, resilience, and systems-control perspectives into a temporally structured framework focused on the evolution of HIT-related risk in routine clinical practice.

The proposed framework should therefore be understood primarily as an integrative, contextual explanatory synthesis of HIT-related risk rather than as a replacement for existing safety or organisational management theories. Its contribution lies in organising established sociotechnical and resilience-oriented concepts into a temporally structured representation of how HIT-related risks evolve, remain hidden, propagate, and become recoverable within routine digitally mediated clinical work.

### 6.4 Relationship to incident-based learning

This theory also reframes the role of incident reporting and analysis. Rather than viewing incidents solely as discrete events to be classified and counted, the control trajectory perspective treats them as visible endpoints of longer, often invisible processes (2, 67). Incident narratives become valuable not only for identifying contributing factors but also for reconstructing trajectories of emergence, hiddenness, propagation, and recovery (2, 68).

This perspective supports a more cumulative approach to learning from incidents, in which patterns across reports are interpreted as manifestations of underlying sociotechnical dynamics (2, 15, 22). Such an approach is consistent with calls for national and system-level learning from HIT-related incidents, while avoiding the limitations of purely retrospective or harm-focused analyses (2, 69, 70).

### 6.5 Limitations and future research

The Sociotechnical Control Trajectory Theory is intentionally high-level and does not attempt to specify all possible sources of HIT-related risk. Its primary limitation lies in abstraction: while it explains broad patterns, it must be operationalised through empirical research to guide specific interventions. Future research may benefit from integrating the proposed framework with established approaches to technology-induced error analysis and sociotechnical HIT safety evaluation to further refine operational indicators and intervention strategies (61, 62).

Future studies are needed to develop measurable indicators of hiddenness, propagation, and recoverability, and to test the propositions outlined in Section 5 across different clinical domains and health system contexts. Future studies may examine the propositions using prospective safety assessment approaches, including HFMEA and related risk analysis methods, alongside traditional incident-based investigations. Future research should also examine the extent to which the proposed trajectory is observable across different healthcare settings, technologies, and risk assessment methods, including both incident-based and prospective safety studies.

Additionally, while the theory is grounded in extensive incident-based research, it does not directly capture patient perspectives. Integrating patient experience data and outcomes into control trajectory analyses represents an important avenue for future work. The theory also does not provide a fully operationalised mechanism for direct pre-harm detection of latent HIT-related risk. Instead, it proposes a conceptual basis for identifying indirect sociotechnical indicators that may support earlier recognition of emerging control degradation.

## 7 Conclusion

By conceptualising HIT-related risk as a sociotechnical control trajectory, this article offers a unifying explanation for why digital health technologies continue to produce unexpected safety challenges despite advances in system design and incident reporting. Rather than proposing a wholly new safety paradigm, this article presents an integrative, HIT-specific explanatory framework that brings together established sociotechnical, resilience, systems control, and patient safety perspectives to explain the evolution of HIT-related risk. The theory integrates empirical insights from incident-based research into a dynamic, mechanism-oriented framework that emphasises emergence, hiddenness, propagation, and recoverability. The framework is particularly intended to support understanding of digitally mediated and interoperable clinical environments in which risks may remain latent, propagate rapidly across workflows, and exceed the explanatory scope of many conventional organisational safety-management approaches. In doing so, it provides a foundation for more proactive, system-level approaches to managing risk in increasingly complex digital health ecosystems.

## References

[1] Palojoki S Mäkelä M Lehtonen L Saranto K. An analysis of electronic health record–related patient safety incidents. Health Informatics J. (2016) 23(2):134–45. 10.1177/1460458216631072 26951568

[2] Jabin MSR. Identifying and Characterising Problems Arising from Interactions Between Medical Imaging and Health Information Technology as a Basis for Improvements in Practice. Adelaide: University of South Australia (2019).

[3] Jabin MSR Hammar T. Issues with the Swedish e-prescribing system – an analysis of health information technology-related incident reports using an existing classification system. Digit Health. (2022) 8:20552076221131139. 10.1177/20552076221131139 36249479 PMC9554230

[4] Jabin MSR. Health information technology–related loss of central surveillance data in a heart intensive care unit: multi-framework case report. JMIR Hum Factors. (2026) 13:e92560. 10.2196/92560 41955001 PMC13064593

[5] Jabin MSR Pan D. Software-related challenges in Swedish healthcare through the lens of incident reports: a desktop study. Digit Health. (2023) 9:20552076231203600. 10.1177/20552076231203600 37744748 PMC10515578

[6] Jabin MSR Steen M Wepa D Bergman P. Assessing the healthcare quality issues for digital incident reporting in Sweden: incident reports analysis. Digit Health. (2023) 9:20552076231174307. 10.1177/20552076231174307 37188073 PMC10176549

[7] Jabin MSR Pan D Nilsson E. Characterizing healthcare incidents in Sweden related to health information technology affecting care management of multiple patients. Health Informatics J. (2022) 28(2):14604582221105440. 10.1177/14604582221105440 35762538

[8] Jabin MSR Pan D Nilsson E. Characterizing patient details-related challenges from health information technology-related incident reports from Swedish healthcare. Front Digit Health. (2024) 6:1260521. 10.3389/fdgth.2024.1260521 38380372 PMC10876894

[9] Jabin MSR Wepa D Hassoun A. A case report of system configuration issue in medical imaging due to system upgrade- changes in hardware and software. Front Digit Health. (2024) 6:1371761. 10.3389/fdgth.2024.1371761 39347445 PMC11427759

[10] Jabin MSR. Operational disruption in healthcare associated with software functionality issue due to software security patching: a case report. Front Digit Health. (2024) 6:1367431. 10.3389/fdgth.2024.1367431 38550716 PMC10973543

[11] Pan D Nilsson E Rahman Jabin MS. A review of incidents related to health information technology in Swedish healthcare to characterise system issues as a basis for improvement in clinical practice. Health Informatics J. (2024) 30(3):14604582241270742. 10.1177/14604582241270742 39116887

[12] Jabin MSR. The need for a refined classification system and national incident reporting system for health information technology-related incidents. Front Digit Health. (2024) 6. 10.3389/fdgth.2024.1422396 PMC11310167 39131183

[13] Jabin MSR. Why health information technology safety problems remain invisible. Front Digit Health. (2026) 8. 10.3389/fdgth.2026.1785141 PMC13219026 42221162

[14] Jabin MSR. Why digital health fails silently: a sociotechnical theory of health information technology–related risk. Front Digit Health. (2026) 8. 10.3389/fdgth.2026.1785086 PMC13231400 42245003

[15] Sittig DF Singh H. A new sociotechnical model for studying health information technology in complex adaptive healthcare systems. Qual Saf Health Care. (2010) 19(Suppl 3):i68–74. 10.1136/qshc.2010.042085 20959322 PMC3120130

[16] Sittig DF Singh H. Defining health information technology-related errors: new developments since to err is human. Arch Intern Med. (2011) 171(14):1281–4. 10.1001/archinternmed.2011.327 21788544 PMC3677061

[17] Friedman CP Lomotan EA Richardson JE Ridgeway JL. Socio-technical infrastructure for a learning health system. Learning Health Systems. (2024) 8(1):e10405. 10.1002/lrh2.10405 38249851 PMC10797563

[18] Livesay K Petersen S Walter R Zhao L Butler-Henderson K Abdolkhani R. Sociotechnical challenges of digital health in nursing practice during the COVID-19 pandemic: national study. JMIR Nursing. (2023) 6:e46819. 10.2196/46819 37585256 PMC10468699

[19] Walsh K Antony J. An assessment of quality costs within electronic adverse incident reporting and recording systems. Int J Health Care Qual Assur. (2009) 22(3):203–20. 10.1108/09526860910953494 19537183

[20] Lewis AE Weiskopf N Abrams ZB Foraker R Lai AM Payne PRO. Electronic health record data quality assessment and tools: a systematic review. J Am Med Inform Assoc. (2023) 30(10):1730–40. 10.1093/jamia/ocad120 37390812 PMC10531113

[21] Blijleven V Hoxha F Jaspers M. Workarounds in electronic health record systems and the revised sociotechnical electronic health record workaround analysis framework: scoping review. J Med Internet Res. (2022) 24(3):e33046. 10.2196/33046 35289752 PMC8965666

[22] Lee SM Lee D. Effects of healthcare quality management activities and sociotechnical systems on internal customer experience and organizational performance. Serv Bus. (2022) 16(1):1–28. 10.1007/s11628-022-00478-9

[23] Ndabu T Mulgund P Sharman R Singh R. Perceptual gaps between clinicians and technologists on health information technology-related errors in hospitals: observational study. JMIR Hum Factors. (2021) 8(1):e21884. 10.2196/21884 33544089 PMC7971770

[24] Senders JW Moray N. Human Error: Cause, Prediction and Reduction. Hillsdale, NJ: Lawrence Erlbaum Associates (1991).

[25] La Pietra L. Medical errors and clinical risk management: state of the art. Acta Otorhinolaryngol Ital. (2005) 25(6):339. 16749601 PMC2639900

[26] Hollnagel E Wears RL Braithwaite J. From Safety-I to Safety-II: A White Paper. Odense: The Resilient Health Care Net (2015). Published simultaneously by the University of Southern Denmark, University of Florida, USA, and Macquarie University, Australia.

[27] Braithwaite J Wears RL Hollnagel E. Resilient health care: turning patient safety on its head. Int J Qual Health Care. (2015) 27(5):418–20. 10.1093/intqhc/mzv063 26294709

[28] Reason J. Managing the Risks of Organizational Accidents. Abingdon: Routledge (2016).

[29] Reason J. Human error: models and management. Br Med J. (2000) 320(7237):768–70. 10.1136/bmj.320.7237.768 10720363 PMC1117770

[30] Sittig DF Singh H. Electronic health records and national patient-safety goals. N Engl J Med. (2012) 367(19):1854–60. 10.1056/NEJMsb1205420 23134389 PMC3690003

[31] Ash JS Sittig DF Dykstra RH Guappone K Carpenter JD Seshadri V. Categorizing the unintended sociotechnical consequences of computerized provider order entry. Int J Med Inform. (2007) 76(Suppl 1):S21–7. 10.1016/j.ijmedinf.2006.05.017 16793330

[32] Magrabi F Ong MS Runciman W Coiera E. Patient safety problems associated with heathcare information technology: an analysis of adverse events reported to the US food and drug administration. AMIA Annu Symp Proc. (2011) 2011:853–7. 22195143 PMC3243129

[33] Magrabi F Ong M-S Runciman W Coiera E. Using FDA reports to inform a classification for health information technology safety problems. J Am Med Inform Assoc. (2012) 19(1):45–53. 10.1136/amiajnl-2011-000369 21903979 PMC3240763

[34] McDermott O Antony J Sony M Rosa A Hickey M Grant TA. A study on Ishikawa’s original basic tools of quality control in healthcare. TQM J. (2023) 35(7):1686–705. 10.1108/TQM-06-2022-0187

[35] Kawu AA Hederman L Doyle J O’Sullivan D. Patient generated health data and electronic health record integration, governance and socio-technical issues: a narrative review. Inform Med Unlocked. (2023) 37:101153. 10.1016/j.imu.2022.101153

[36] Coiera E Magrabi F. Information system safety. In: Hoyt RE, Yoshihashi A, editors. Guide to Health Informatics. Boca Raton, FL, USA: CRC Press, Taylor & Francis Group (2015).

[37] Magrabi F. Identifying patient safety problems associated with information technology in general practice: an analysis of incident reports. BMJ Qual Saf. (2015) 24:870–80. 10.1136/bmjqs-2015-004323 26543068

[38] Smith MW Ash JS Sittig DF Singh H. Resilient practices in maintaining safety of health information technologies. J Cogn Eng Decis Mak. (2014) 8(3):265–82. 10.1177/1555343414534242 25866492 PMC4361460

[39] Leveson NG. Engineering a Safer World: Systems Thinking Applied to Safety. Cambridge: The MIT Press (2012).

[40] Hollnagel E, Safety-II in Practice: Developing the Resilience Potentials. Boca Raton: CRC Press (2017). p. 1–130.

[41] Sittig DF Wright A Coiera E Magrabi F Ratwani R Bates DW. Current challenges in health information technology–related patient safety. Health Informatics J. (2020) 26(1):181–9. 10.1177/1460458218814893 30537881 PMC7510167

[42] Ross TK. Health Care Quality Management: Tools and Applications. Hoboken: John Wiley & Sons (2013).

[43] Singh H Sittig DF. Measuring and improving patient safety through health information technology: the health IT safety framework. BMJ Qual Saf. (2016) 25(4):226–32. 10.1136/bmjqs-2015-004486 26369894 PMC4819641

[44] Castro GM Buczkowski L Hafner JM. The contribution of sociotechnical factors to health information technology-related sentinel events. Jt Comm J Qual Patient Saf. (2016) 42(2):70–6. 10.1016/s1553-7250(16)42008-8 26803035

[45] Harrison MI Koppel R Bar-Lev S. Unintended consequences of information technologies in health care–an interactive sociotechnical analysis. J Am Med Inform Assoc. (2007) 14(5):542–9. 10.1197/jamia.M2384 17600093 PMC1975796

[46] Aarts J. Towards safe electronic health records: a socio-technical perspective and the need for incident reporting. Health Policy Technol. (2012) 1(1):8–15. 10.1016/j.hlpt.2012.01.008

[47] Coiera E Aarts J Kulikowski C. The dangerous decade. J Am Med Inform Assoc. (2011) 19(1):2–5. 10.1136/amiajnl-2011-000674 22116642 PMC3240771

[48] Coiera E. Why e-health is so hard. Med J Aust. (2013) 198(4):178–9. 10.5694/mja13.10101 23451947

[49] Dafallah A Witter S. Diaspora as partners: strengthening resilience of health systems and communities amidst aid volatility. BMJ Glob Health. (2025) 10(6):e019622. 10.1136/bmjgh-2025-019622 40537273 PMC12182014

[50] Pilosof NP Welcman Y Barrett M Oborn E Barrett S. Building digital resilience: leading healthcare transformation through an online community. Front Digit Health. (2025) 7:2025. 10.3389/fdgth.2025.1656804 PMC12446309 40978698

[51] Marx DA Slonim AD. Assessing patient safety risk before the injury occurs: an introduction to sociotechnical probabilistic risk modelling in health care. Qual Saf Health Care. (2003) 12(Suppl 2):ii33–8. 10.1136/qhc.12.suppl_2.ii33 14645893 PMC1765773

[52] Saleem JJ Russ AL Sanderson P Johnson TR Zhang J Sittig DF. Current challenges and opportunities for better integration of human factors research with development of clinical information systems. Yearb Med Inform. (2009) 18:48–58. 10.1055/s-0038-1638638 19855872

[53] Sittig DF Flanagan T Sengstack P Cholankeril RT Ehsan S Heidemann A. Revisions to the safety assurance factors for electronic health record resilience (SAFER) guides to update national recommendations for safe use of electronic health records. J Am Med Inform Assoc. (2025) 32(4):755–60. 10.1093/jamia/ocaf018 40220287 PMC12005625

[54] Sittig DF Wright A Coiera E Magrabi F Ratwani R Bates DW. Current challenges in health information technology–related patient safety. Health Informatics J. (2018) 26(1):181–9. 10.1177/1460458218814893 30537881 PMC7510167

[55] Woods DD. Four concepts for resilience and the implications for the future of resilience engineering. Reliab Eng Syst Saf. (2015) 141:5–9. 10.1016/j.ress.2015.03.018

[56] Perrow C. Normal Accidents - Living with High Risk Technologies. New York: Basic Books (1984).

[57] Coiera E Magrabi F Talmon J. Engineering technology resilience through informatics safety science. J Am Med Inform Assoc. (2017) 24(2):244–5. 10.1093/jamia/ocw162 28040683 PMC7651896

[58] Sittig DF Gonzalez D Singh H. Contingency planning for electronic health record-based care continuity: a survey of recommended practices. Int J Med Inform. (2014) 83(11):797–804. 10.1016/j.ijmedinf.2014.07.007 25200197

[59] Magrabi F Aarts J Nohr C Baker M Harrison S Pelayo S. A comparative review of patient safety initiatives for national health information technology. Int J Med Inform. (2013) 82(5):e139–48. 10.1016/j.ijmedinf.2012.11.014 23266061

[60] Sittig DF Wright A Simonaitis L Carpenter JD Allen GO Doebbeling BN. The state of the art in clinical knowledge management: an inventory of tools and techniques. Int J Med Inform. (2010) 79(1):44–57. 10.1016/j.ijmedinf.2009.09.003 19828364 PMC2895508

[61] Borycki EM Kushniruk AW Keay L Kuo A. A framework for diagnosing and identifying where technology-induced errors come from. Stud Health Technol Inform. (2009) 148:181–7. 19745249

[62] Sittig DF Singh H. A sociotechnical approach to electronic health record related safety. In: Leviss J, editor. Key Advances in Clinical Informatics. Amsterdam: Academic Press (2017). p. 197–216.

[63] Kaplan RS Norton DP. The Balanced Scorecard: Translating Strategy into Action. Boston: Harvard Business Review Press (1996).

[64] Kotter JP. Leading Change. Boston: Harvard Business School Press (1996).

[65] The Evaluation Support Team. The Theory of Change Process – Guidance for Outcome Delivery Plans. Edinburgh: The Evaluation Support Scotland (2021).

[66] Hollnagel E. Resilience Engineering in Practice: A Guidebook. Farnham: Ashgate Publishing (2010).

[67] Pham JC Girard T Pronovost PJ. What to do with healthcare incident reporting systems. J Public Health Res. (2013) 2(3):e27. 10.4081/jphr.2013.e27 25170498 PMC4147750

[68] Mandel C Runciman W. System for reporting and analysing incidents. In: Brady Z Partridge M, editors. Radiological Safety and Quality: Paradigms in Leadership and Innovation. Berlin: Springer (2014). p. 203–21.

[69] Hannaford N Mandel C Crock C Buckley K Magrabi F Ong M. Learning from incident reports in the Australian medical imaging setting: handover and communication errors. Br J Radiol. (2013) 86(1022):20120336. 10.1259/bjr.20120336 23385994 PMC3608041

[70] Vincent C. Incident reporting and patient safety. BMJ. (2007) 334:51. 10.1136/bmj.39071.441609.80 17218667 PMC1767279

